# Supplementary material for: Integrated genomics and comprehensive validation reveal drivers of genomic evolution in esophageal adenocarcinoma
Source: Commun Biol. 2021 May 24;4:617. doi: 10.1038/s42003-021-02125-x (PMC8144613; doi:10.1038/s42003-021-02125-x)
Supplement: Supplementary file 2 — Supplementary Information [file 42003_2021_2125_MOESM2_ESM.pdf]

SUPPLEMENTARY MATERIAL:

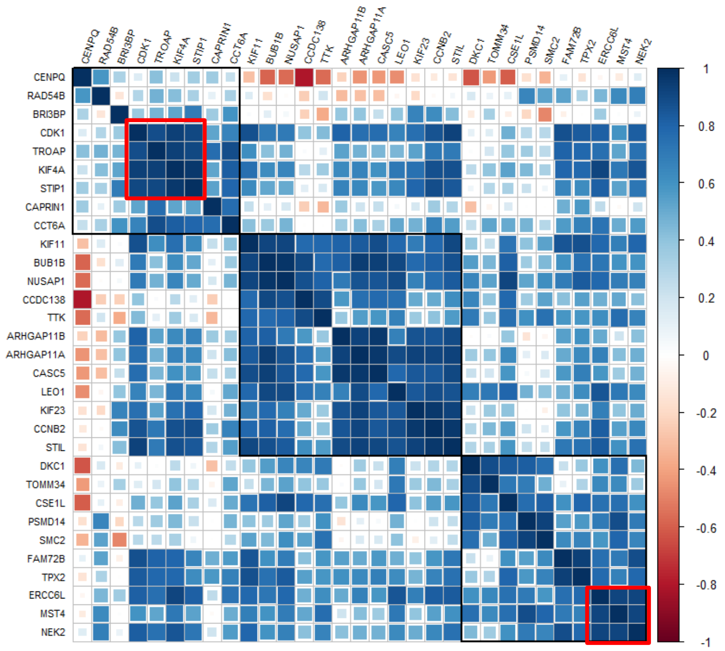

Supplementary Figure 1. Co-upregulated GIS31 genes in EAC. GIS31 genes in EAC patient dataset (from TCGA) were analyzed by a pairwise correlation of their expression, using Pearson method.

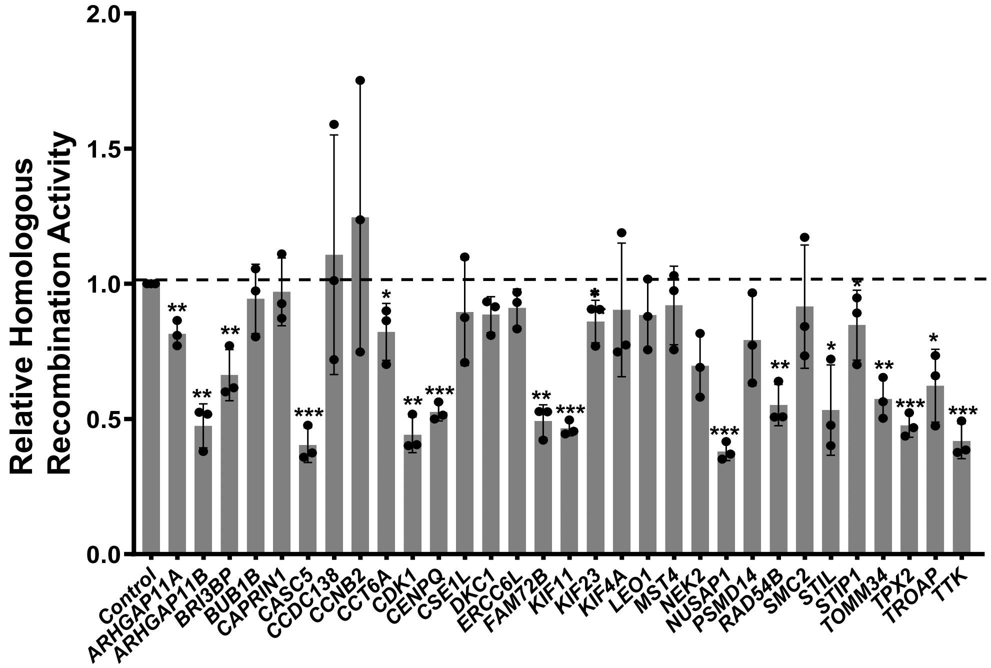

Supplementary Figure 2. Functional siRNA screen evaluating GIS31 genes for impact on homologous recombination (HR) activity in EAC cells. EAC (FLO1) cells were transfected with siRNAs, either control (non-targeting) or those targeting 31 potential genomic instability (GIS31) genes, and impact on homologous recombination assessed using strand exchange assay described in Methods section. Bar graphs show percent inhibition of homologous recombination activity; error bars represent SDs of three independent experiments. Two-tailed p-values, indicating significance of difference relative to control siRNA-transfected cells, are shown as: * < 0.05 – > 0.005; ** < 0.005 – > 0.0001; *** < 0.0001 – < 0.000005.

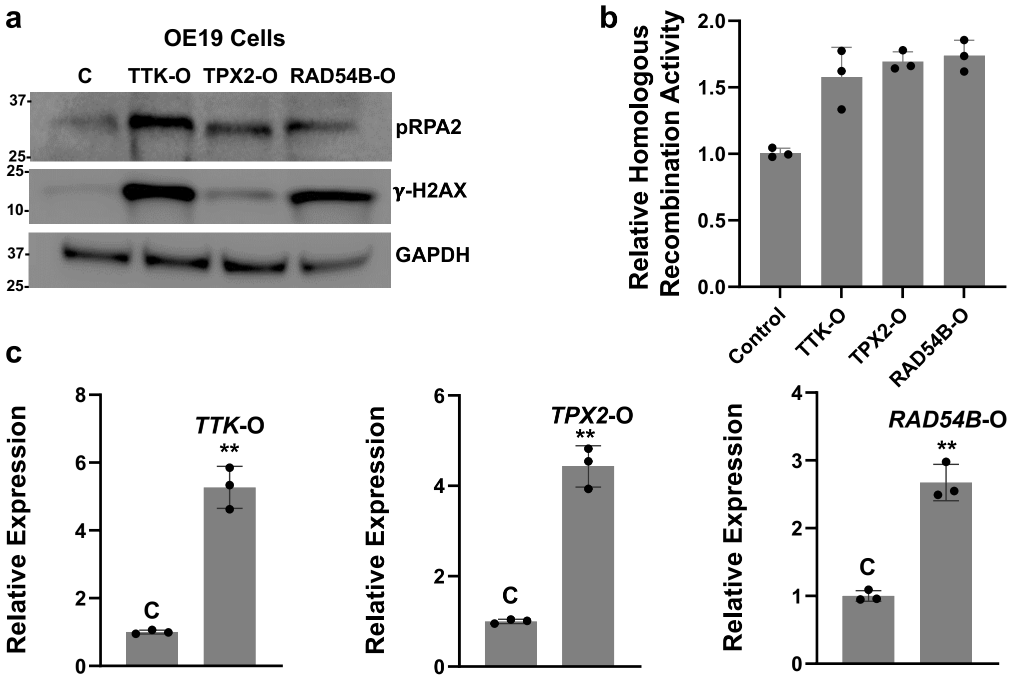

Supplementary Figure 3. Overexpression of *TTK*, *TPX2* and *RAD54B* increases DNA breaks and homologous recombination activity in EAC (OE19) cells. (a-b) OE19 cells were transfected with control plasmid (C) or those overexpressing *TTK* (*TTK*-O), *TPX2* (*TPX2*-O) or *RAD54B* (*RAD54B*-O), selected in puromycin and evaluated for γ-H2AX and phosphorylated-RPA32, using Western blotting (a), and homologous recombination activity, using a plasmid-based assay (b). Error bars indicate SDs of experiments conducted in triplicate; Two-tailed p values: = p < 0.5; (**c)** The transgene overexpression confirmed by Q-PCR.

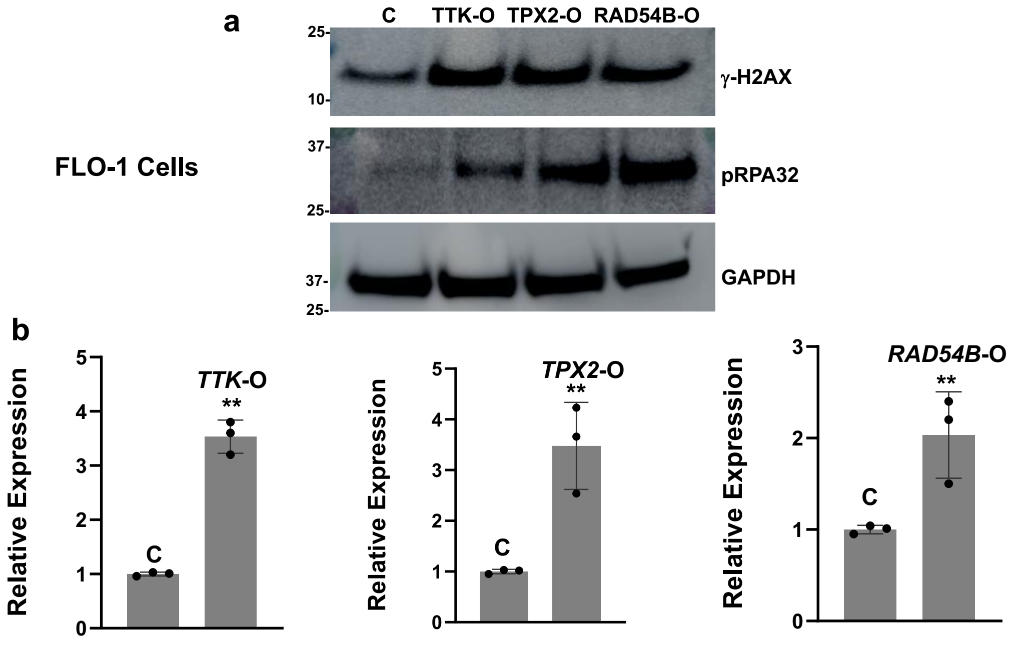

Supplementary Figure 4. Overexpression of *TTK*, *TPX2* and *RAD54B* increases DNA breaks and DNA end resection in EAC (FLO-1) cells. a) FLO-1 cells were transfected with control plasmid (C) or those overexpressing *TTK* (*TTK*-O), *TPX2* (*TPX2*-O) or *RAD54B* (*RAD54B*-O), selected in puromycin and evaluated for γ-H2AX and phosphorylated-RPA32, using Western blotting; **b)** The transgene overexpression confirmed by Q-PCR.

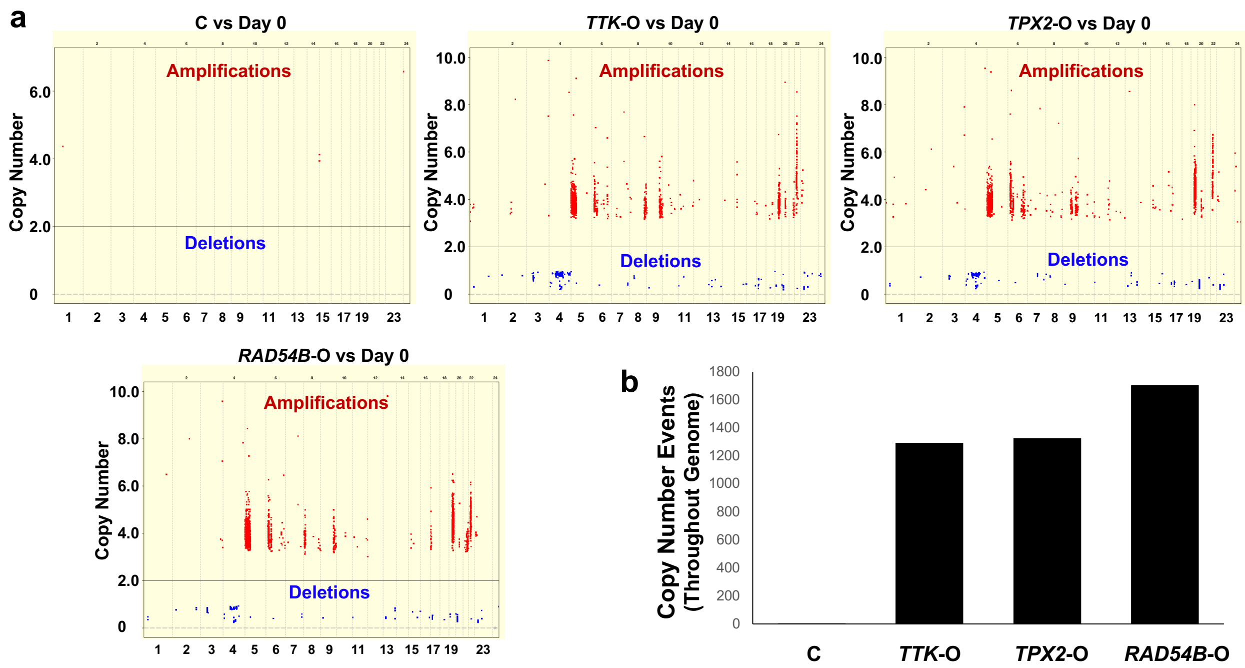

Supplementary Figure 5. Overexpression of *TTK*, *TPX2* and *RAD54B* in normal primary esophageal cells induces the acquisition of new copy number changes over time. Normal primary human esophageal epithelial cells (HEsEpi; ScienCell) were transfected with control plasmid (C) or those overexpressing *TTK* (*TTK*-O), *TPX2* (*TPX2*-O) or *RAD54B* (*RAD54B*-O), selected in puromycin and cultured for thirty days. DNA from these and baseline control (day 0) cells was purified and acquisition of copy number events during growth of cells in culture vs. day 0 cells (representing baseline genome) monitored, using SNP6.0 arrays (Affymetrix); a copy event was defined as a change in ≥ 5 consecutive CNV probes by 1 copy. **(a)** Images showing copy number events, as red (amplification) and blue (deletion) dots on all chromosomes, in cultured relative to baseline (day 0) cells; **(b)** Bar graph showing total copy-number change events, throughout genome.

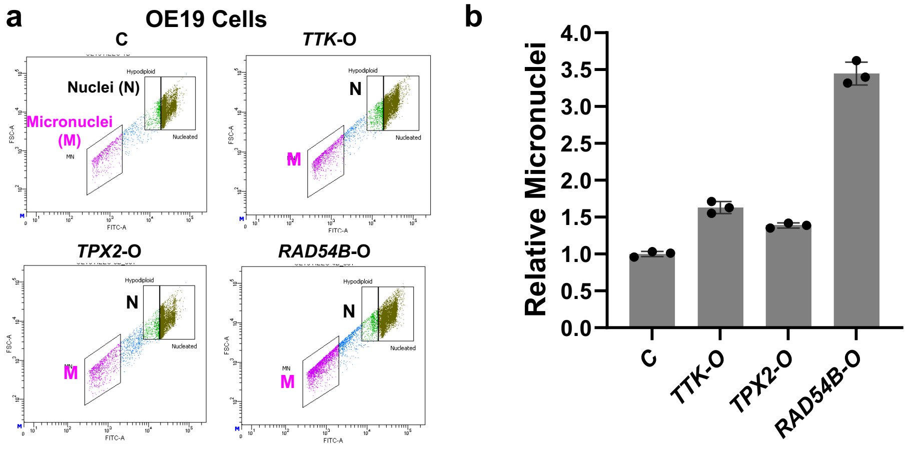

Supplementary Figure 6. Overexpression of *TTK*, *TPX2* and *RAD54B* increases genomic instability in EAC (OE19) cells. OE19 cells were transfected with control plasmid (C) or those overexpressing *TTK* (*TTK*-O), *TPX2* (*TPX2*-O) or *RAD54B* (*RAD54B*-O), selected in puromycin and evaluated for micronuclei, a marker of genomic instability. Flow cytometry images of micronuclei (a) and bar graphs showing percentage of micronuclei (b) are shown.

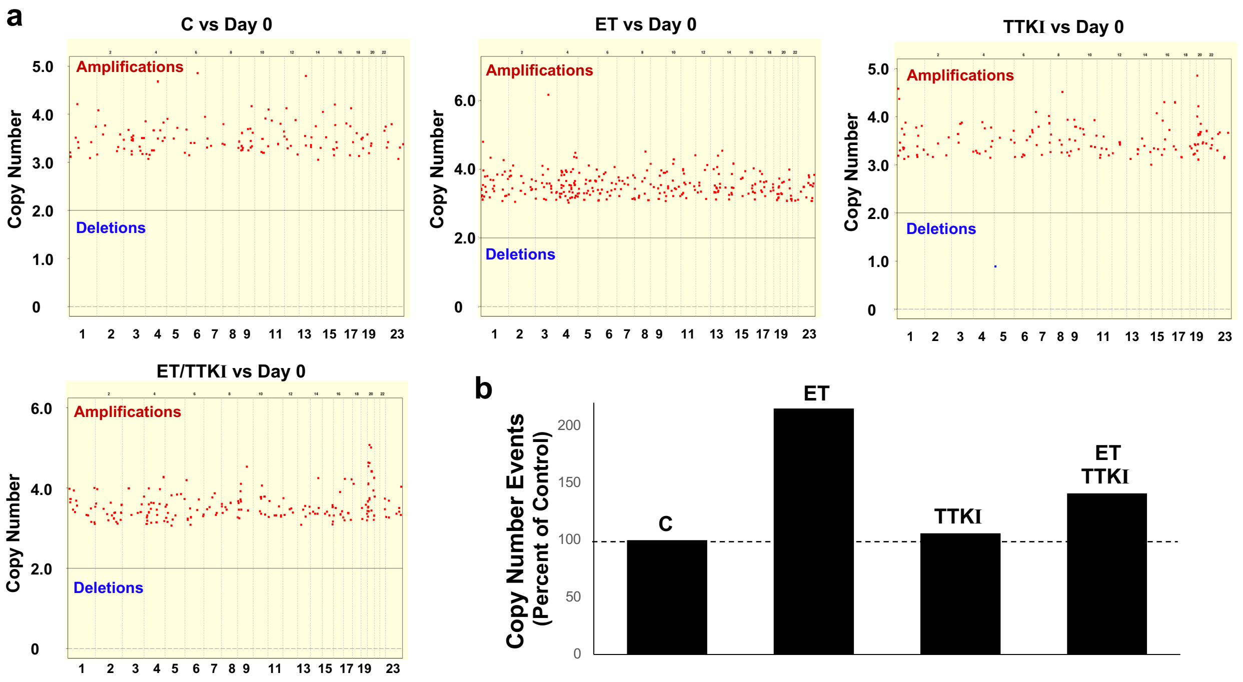

Supplementary Figure 7. TTK inhibitor reduces etoposide-induced acquisition of copy number events in EAC cells. FLO-1 cells, control (C; DMSO only) or those treated with TTK inhibitor (TTK-I; 10 nM) and etoposide (ET; 1 μM), alone as well as in combination with each other for 3 weeks. DNA from these and baseline control (day 0) cells was purified and acquisition of copy number events during growth of cells in culture vs. day 0 cells (representing baseline genome) monitored, using Axiom™ Precision Medicine Diversity Arrays; a copy event was defined as a change in ≥ 3 consecutive CNV probes by 1 copy. **(a)** Apmlifications (red dots) and deletions (blue dots) on different chromosomes; **(b)** Bar graph showing copy number events throughout genome.

Supplementary Table 1. Known functions of GIS31 genes.

Gene Name	Family	Function/Pathway	Ref. #
ARHGAP11A	Rho GTPase	Cell cycle, DNA damage response	1
ARHGAP11B	Rho GTPase	Brain Development	2
BRI3BP	BRI3 binding protein	Involved in tumorigenesis	3
BUB1B	Serine/Threonine Kinase B	Mitotic checkpoint kinase	4
CAPRIN1	Cell Cycle Associated Protein	Cell proliferation	5
CASC5	Kinetochore protein	Cell cycle regulation	6
CCDC138	Coiled-coil domain-containing	Unknown	
CCNB2	B-type cyclins	G2/M Cell cycle regulation	7
CCT6A	Chaperonin protein	Protein folding	8
CDK1	Cyclin dependent kinase	G2/M Cell cycle regulation	9
CENPQ	Centromere protein	Regulation of mitosis	10
CSE1L	Nuclear export factor	Cell cycle and genomic instability	11
DKC1	Small nucleolar ribonucleoprotein	Cell cycle	12
ERCC6L	Mitotic helicase	Mitosis checkpoint regulation	13
FAM72B	Family with sequence similarity 72	Cell cycle regulation	14
KIF11	Kinetochore associated protein	Mitosis checkpoint regulation	15
KIF23	Kinetochore associated protein	Mitosis checkpoint regulation	15
KIF4A	Kinetochore associated protein	Mitosis checkpoint regulation	15
LEO1	RNA polymerase II associated factor Paf1	Oncogene	16
MST4	Serine/threonine protein kinase	Promote cell growth and transformation	17
NEK2	Mitotic kinase	Cancer progression	18
NUSAP1	Nucleolar & spindle associated	Promote cancer progression	19
PSMD14	Deubiquitinating enzyme	Promote tumor metastasis	20
RAD54B	DNA repair and recombination protein	Promotes homologous recombination	21
SMC2	Structural maintenance of chromosomes protein	Involved in DNA repair pathway and genomic instability	22
STIL	Centriolar replication factor	Involved in DNA damage response	23
STIP1	Stress induced phosphoprotein	Tumor growth, metastasis	24
TOMM34	Mitochondrial import receptor	Promotes cancer growth	25
TPX2	Microtubule-associated protein	Genomic instability, cancer	26
TROAP	Cytoskeleton, spindle assembly	Cancer and metastasis	27
TTK	Mitotic kinase	Homologous recombination and cancer growth	28

SUPPLEMENTARY REFERENCES:

1. Kagawa Y, Matsumoto S, Kamioka Y, Mimori K, Naito Y, Ishii T, Okuzaki D, Nishida N, Maeda S, Naito A, Kikuta J, Nishikawa K, Nishimura J, Haraguchi N, Takemasa I, Mizushima T, Ikeda M, Yamamoto H, Sekimoto M, Ishii H, Doki Y, Matsuda M, Kikuchi A, Mori M, Ishii M. Cell cycle-dependent Rho GTPase activity dynamically regulates cancer cell motility and invasion in vivo. PLoS One. 2013 Dec 30;8(12):e83629. doi: 10.1371/journal.pone.0083629. eCollection 2013. PMID: 24386239

2. Florio M, Albert M, Taverna E, Namba T, Brandl H, Lewitus E, Haffner C, Sykes A, Wong FK, Peters J, Guhr E, Klemroth S, Prüfer K, Kelso J, Naumann R, Nüsslein I, Dahl A, Lachmann R, Pääbo S, Huttner WB. Human-specific gene ARHGAP11B promotes basal progenitor amplification and neocortex expansion. Science. 2015 Mar 27; 347(6229):1465-70.

3. Ha SA, Shin S.M., Lee Y.J., Kim S., Kim H.K., Namkoong H., Lee H., Lee Y.S., Cho Y.S., Park Y.G., Jeon H.M., Oh C., Kim J.W. HCCRBP-1 directly interacting with HCCR-1 induces tumorigenesis through P53 stabilization Int. J. Cancer 122:501-508(2008)

4. Hanks S, Coleman K, Reid S, Plaja A, Firth H, Fitzpatrick D, Kidd A, Méhes K, Nash R, Robin N, Shannon N, Tolmie J, Swansbury J, Irrthum A, Douglas J, Rahman N. Constitutional aneuploidy and cancer predisposition caused by biallelic mutations in BUB1B. Nat Genet. 2004 Nov;36(11):1159-61.

5. Wang, B., David, M. D., Schrader, J. W. Absence of Caprin-1 results in defects in cellular proliferation. J. Immun. 175: 4274-4282, 2005.

6. Genin, A., Desir, J., Lambert, N., Biervliet, M., Van Der Aa, N., Pierquin, G., Killian, A., Tosi, M., Urbina, M., Lefort, A., Libert, F., Pirson, I., Abramowicz, M. Kinetochore KMN network gene CASC5 mutated in primary microcephaly. Hum. Molec. Genet. 21: 5306-5317, 2012.

7. Nam, H.-J., van Deursen, J. M. Cyclin B2 and p53 control proper timing of centrosome separation. Nature Cell Biol. 16: 535-546, 2014.

8. Li WZ, Lin P, Frydman J, Boal TR, Cardillo TS, Richard LM, Toth D, Lichtman MA, Hartl FU, Sherman F (August 1994). "Tcp20, a subunit of the eukaryotic TRiC chaperonin from humans and yeast". J Biol Chem. 269 (28): 18616–22. PMID 8034610

9. Santamaría D, Barrière C, Cerqueira A, et al. Cdk1 is sufficient to drive the mammalian cell cycle. Nature. 2007;448(7155):811-815. doi:10.1038/nature06046

10. Okada, M., Cheeseman, I. M., Hori, T., Okawa, K., McLeod, I. X., Yates, J. R., III, Desai, A., Fukagawa, T. The CENP-H-I complex is required for the efficient incorporation of newly synthesized CENP-A into centromeres. Nature Cell Biol. 8: 446-457, 2006.

11. Wellmann, A., Flemming, P., Behrens, P., Wuppermann, K., Lang, H., Oldhafer, K., Pastan, I., Brinkmann, U. High expression of the proliferation and apoptosis associated CSE1L/CAS gene in hepatitis and liver neoplasms: correlation with tumor progression. Int. J. Mol. Med. (2001)

12. Heiss NS, Knight SW, Vulliamy TJ, et al. X-linked dyskeratosis congenita is caused by mutations in a highly conserved gene with putative nucleolar functions. Nat Genet. 1998;19(1):32-38

13. Baumann, C., Korner, R., Hofmann, K., Nigg, E. A. PICH, a centromere-associated SNF2 family ATPase, is regulated by Plk1 and required for the spindle checkpoint. Cell 128: 101-114, 2007.

14. Rahane CS, Kutzner A, Heese K. A cancer tissue-specific FAM72 expression profile defines a novel glioblastoma multiform (GBM) gene-mutation signature. J Neurooncol. 2019;141(1):57-70.

15. Liu X, Gong H, Huang K. Oncogenic role of kinesin proteins and targeting kinesin therapy Cancer Sci. 2013 Jun;104(6):651-6.

16. Chong PS, Zhou J, Cheong LL, et al. LEO1 is regulated by PRL-3 and mediates its oncogenic properties in acute myelogenous leukemia. Cancer Res. 2014;74(11):3043-3053. doi:10.1158/0008-5472.CAN-13-2321

17. Ma X, Zhao H, Shan J, Long F, Chen Y, Chen Y, Zhang Y, Han X, Ma D (2007). "PDCD10 interacts with Ste20-related kinase MST4 to promote cell growth and transformation via modulation of the ERK pathway". Mol. Biol. Cell. 18 (6): 1965–78. doi:10.1091/mbc.E06-07-0608

18. Fang Y, Zhang X. Targeting NEK2 as a promising therapeutic approach for cancer treatment. Cell Cycle. 2016;15(7):895-907. doi:10.1080/15384101.2016.1152430
19. Gordon CA, Gong X, Ganesh D, Brooks JD. NUSAP1 promotes invasion and metastasis of prostate cancer. Oncotarget. 2017;8(18):29935-29950. doi:10.18632/oncotarget.15604

20. Zhu R, Liu Y, Zhou H, et al. Deubiquitinating enzyme PSMD14 promotes tumor metastasis through stabilizing SNAIL in human esophageal squamous cell carcinoma. Cancer Lett. 2018;418:125-134. doi:10.1016/j.canlet.2018.01.025

21. Yasuhara T, Suzuki T, Katsura M, Miyagawa K. Rad54B serves as a scaffold in the DNA damage response that limits checkpoint strength. Nat Commun. 2014;5:5426.

22. Wu N, Yu H. The Smc complexes in DNA damage response. Cell Biosci. 2012;2:5. Published 2012 Feb 27. doi:10.1186/2045-3701-2-5

23. Rabinowicz N, Mangala LS, Brown KR, et al. Targeting the centriolar replication factor STIL synergizes with DNA damaging agents for treatment of ovarian cancer. Oncotarget. 2017;8(16):27380-27392. doi:10.18632/oncotarget.16068

24. Sun X, Cao N, Mu L, Cao W. Stress induced phosphoprotein 1 promotes tumor growth and metastasis of melanoma via modulating JAK2/STAT3 pathway. Biomed Pharmacother. 2019;116:108962. doi:10.1016/j.biopha.2019.108962

25. Muller P, Coates PJ, Nenutil R, et al. Tomm34 is commonly expressed in epithelial ovarian cancer and associates with tumour type and high FIGO stage. J Ovarian Res. 2019;12(1):30. Published 2019 Mar 27. doi:10.1186/s13048-019-0498-0

26. van Gijn SE, Wierenga E, van den Tempel N, et al. TPX2/Aurora kinase A signaling as a potential therapeutic target in genomically unstable cancer cells. Oncogene. 2019;38(6):852-867. doi:10.1038/s41388-018-0470-2

27. Li K, Zhang R, Wei M, et al. TROAP Promotes Breast Cancer Proliferation and Metastasis. Biomed Res Int. 2019;2019:6140951. Published 2019 May 6. doi:10.1155/2019/6140951

28. Chandler BC, Moubadder L, Ritter CL, et al. TTK inhibition radiosensitizes basal-like breast cancer through impaired homologous recombination. J Clin Invest. 2020;130(2):958-973. doi:10.1172/JCI130435
