# Supplementary material for: Integrated genomics and comprehensive validation reveal drivers of genomic evolution in esophageal adenocarcinoma
Source: Commun Biol. 2021 May 24;4:617. doi: 10.1038/s42003-021-02125-x (PMC8144613; doi:10.1038/s42003-021-02125-x)
Supplement: Supplementary file 3 — Description of Additional Supplementary Files [file 42003_2021_2125_MOESM3_ESM.pdf]

Description of Additional Supplementary Files

File name: Supplementary data 1
Description:

Figure 2 (a-b). EAC (FLO1) cells, stably transduced with Cas9, were treated with lentiviral single-guide RNAs, either non-targeting control or those targeting specific genes (two or three guides per gene). Following selection, cells were cultured for 10 days and evaluated for impact on homologous recombination activity (a) and cell viability (b), relative to control (control bar represents average of two different control guides); Error bars represent SDs of three experiments. Two-tailed p values derived by Student's t-Test (*, $p < 0.05 – 0.001$; **, $p < 0.001$) indicate significance of difference relative to control.

Figure 2 (c and f). FLO1 cells were treated with lentiviral constructs, either control or those carrying open reading frames (for overexpression) of genes, selected in puromycin, and evaluated for homologous recombination (c) and micronuclei (a marker of genomic instability) (e-f). (c) Bar graph showing homologous recombination activity, relative to control; Error bars represent SDs of three independent experiments. Two-tailed p values derived by Student's t-Test: *, $p < 0.05$; **, $p < 0.0007$; ***, $p < 0.00005$; (f) Bar graph showing fold change in micronuclei, relative to control cells; Error bars represent SDs of three experiments. * = two-tailed p value derived by Student's t-Test < 0.04.

Figure 3 (a). Gene expression, evaluated in normal primary human esophageal epithelial (HEsEpi) cell and EAC cell lines (FLO-1, OE19 and OE33), using real time PCR; Error bars indicate SDs of experiments conducted in triplicate. Two-tailed p values derived by Student's t-Test: * = $p < 0.05 - 0.001$; ** = $p < 0.001$. **Figure 3 (c and f).** FLO-1 cells were transduced with lentivirus-based shRNAs, either control (CS) or those targeting *TTK*, *TPX2* or *RAD54B*, and following selection, knockdown (KD) of gene expression confirmed by RT PCR (c) and homologous recombination activity measured using a functional assay (f). Error bars in panel c indicate SDs of experiment conducted in triplicate and those in panel f are SDs of three experiments. Two-tailed p values derived by Student's t-Test: * = $p < 0.05 - 0.001$; ** = $p < 0.001$.

Figure 4 (a). Control and knockdown FLO1 cells were cultured and cell viability assessed at various intervals.

Figure 4 (b-d). Control and knockdown FLO-1 cells, cultured for > 10 days in vitro, were subcutaneously injected in SCID mice and tumor growth measured at indicated intervals. Error bars indicate SEMs of tumor sizes in different mice. Two-tailed p values derived by Student's t-Test £ 0.04.

Figure 5 (a and c). Normal primary human esophageal epithelial cells (HEsEpi) were transfected with control plasmid (C) or those overexpressing *TTK* (*TTK-O*), *TPX2* (*TPX2-O*) or *RAD54B* (*RAD54B-O*), selected in puromycin and evaluated for various aspects of genome stability at indicated time points. At day 7 after transfection, the transgene overexpression was confirmed by Q-PCR (a), and cells evaluated for homologous recombination activity, using a plasmid-based assay (c). Error bars indicate SDs of experiments conducted in triplicate. Two-tailed p values derived by Student's t-Test: ** = $p < 0.001$.

Figure 6. (a) Normal or non-cancerous cell types (HEsEpi, primary human esophageal epithelial cells; Het-1A, SV40 large T antigen transfected human esophageal epithelial cells; HDF, human diploid fibroblasts) and EAC cell lines (FLO-1, OE19 AND OE33) were treated with TTK inhibitor (TTK-I, CFI402257) at various concentrations for 72 h and cell viability assessed. Error bars represent SDs of three experiments; **(b)** EAC (FLO-1) cells were injected subcutaneously in SCID mice and following appearance of palpable tumors, mice treated with TTK inhibitor. Tumor volumes were measured at indicated intervals. Error bars represent SDs of tumor sizes from nine control and nine treated mice. **(c-d)** EAC cell lines (FLO-1 and OE19) were treated with inhibitor (TTK-I, CFI402257), alone as well as in the presence of chemotherapeutic agents 5-fluorouracil (c) or cisplatin (d), and cell viability measured after 72 h. Error bars represent SDs of three experiments.

Figure 7 (a). FLO-1 cells were treated with different concentrations of TTK inhibitor (TTK-I, CFI402257) for 48 h and impact on homologous recombination activity evaluated using a plasmid-based assay; Error bars indicate SDs of experiment conducted in triplicate. Two-tailed p values derived by Student's t-Test: ** = $p < 0.001$

Supplementary Figure 2. EAC (FLO1) cells were transfected with siRNAs, either control (non-targeting) or those targeting 31 potential genomic instability (GIS31) genes, and impact on homologous recombination assessed using strand exchange assay described in Methods section. Bar graphs show percent inhibition of homologous recombination activity; error bars represent SDs of three independent experiments. Two-tailed p-values, indicating significance of difference relative to control siRNA-transfected cells, are shown as: * < 0.05 - > 0.005; ** < 0.005 - > 0.0001; *** < 0.0001 - < 0.000005.

Supplementary Figure 3. (b) OE19 cells were transfected with control plasmid (C) or those overexpressing *TTK* (*TTK*-O), *TPX2* (*TPX2*-O) or *RAD54B* (*RAD54B*-O), selected in puromycin and evaluated for homologous recombination activity, using a plasmid-based assay. Error bars indicate SDs of experiments conducted in triplicate; Two-tailed p values: = $p < 0.5$; **(c)** The transgene overexpression confirmed by Q-PCR.

Supplementary Figure 4 (b). FLO-1 cells were transfected with control plasmid (C) or those overexpressing *TTK* (*TTK*-O), *TPX2* (*TPX2*-O) or *RAD54B* (*RAD54B*-O), selected in puromycin and transgene overexpression confirmed by Q-PCR.

Supplementary Figure 6 (b). OE19 cells were transfected with control plasmid (C) or those overexpressing *TTK* (*TTK*-O), *TPX2* (*TPX2*-O) or *RAD54B* (*RAD54B*-O), selected in puromycin and evaluated for micronuclei, a marker of genomic instability. Bar graphs show percentage of micronuclei.

File name: Supplementary data 2

Description: Full blot/gel images.

File name: Supplementary data 3

Description: Clinical information related to esophageal adenocarcinoma (ESCA) dataset GSE19417.

File name: Supplementary data 4

Description: Clinical information related to esophageal adenocarcinoma (ESCA) TCGA dataset.
