# Supplementary material for: Integrated genomics and comprehensive validation reveal drivers of genomic evolution in esophageal adenocarcinoma
Source: Commun Biol. 2021 May 24;4:617. doi: 10.1038/s42003-021-02125-x (PMC8144613; doi:10.1038/s42003-021-02125-x)
Supplement: Supplementary file 5 — Supplementary Data 2 [file 42003_2021_2125_MOESM5_ESM.pptx]

## Slide 1
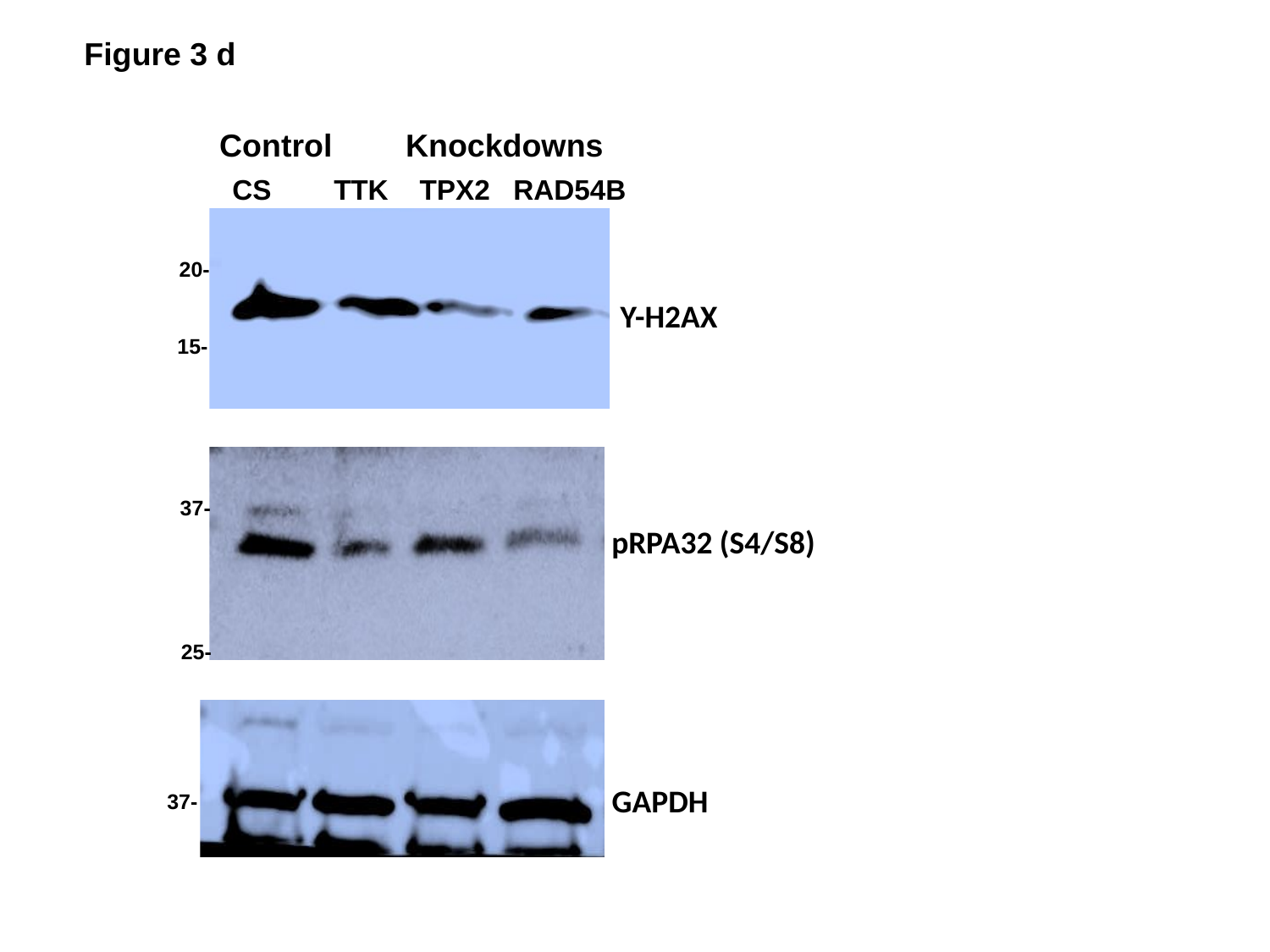

Figure 3 d
Control
Knockdowns
CS TTK TPX2 RAD54B
20-
 15-
Y-H2AX
37-
 25-
pRPA32 (S4/S8)
GAPDH
37-

## Slide 2
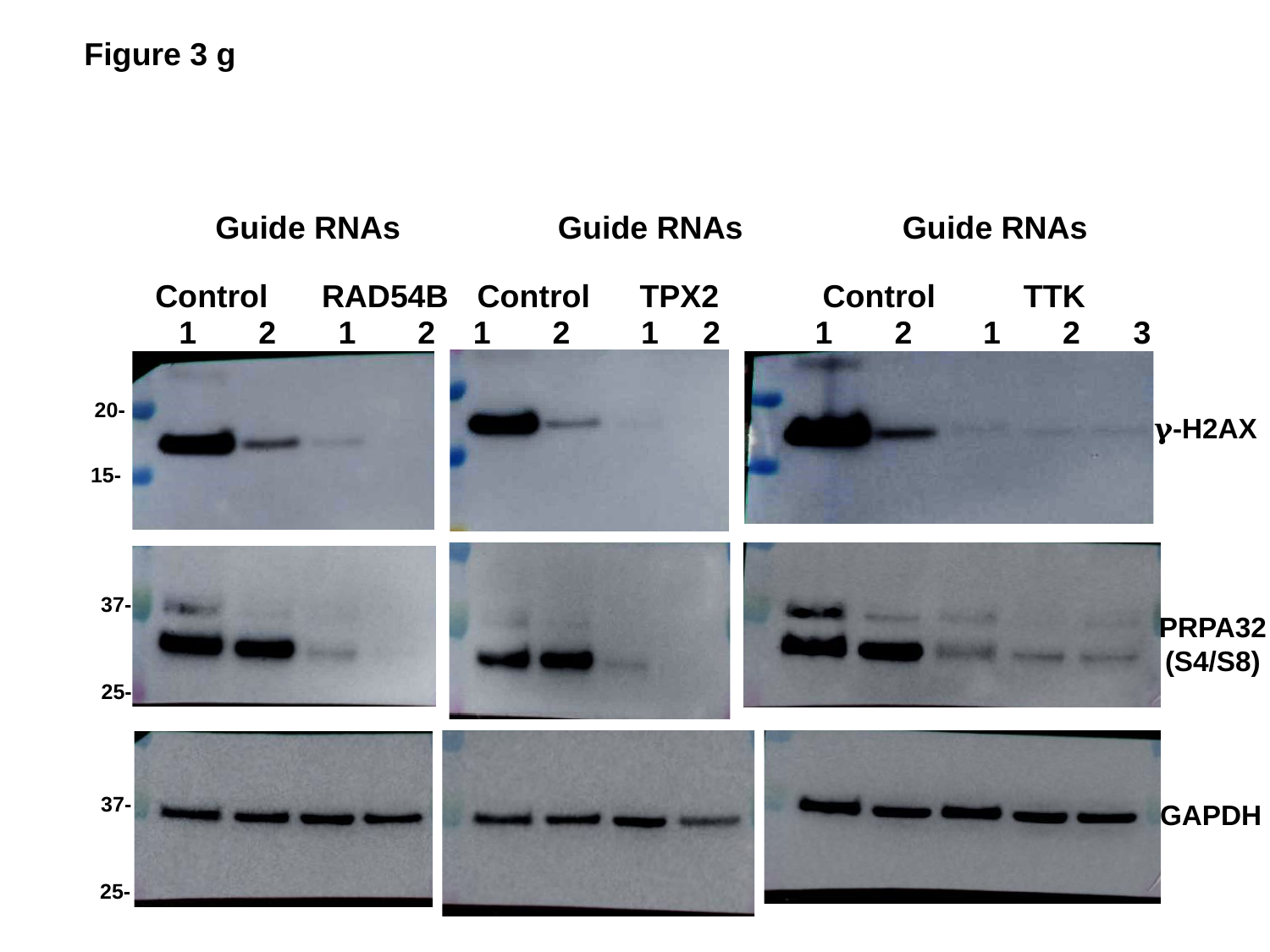

IV
CRISPR/Cas9-mediated knockdown
Figure 3 g
Guide RNAs
Guide RNAs
Guide RNAs
Control
RAD54B
Control
TPX2
Control
TTK
1 2 1 2
1 2 1 2
1 2 1 2 3
20-
 15-
𝛄-H2AX
37-
 25-
PRPA32 (S4/S8)
37-
 25-
GAPDH

## Slide 3
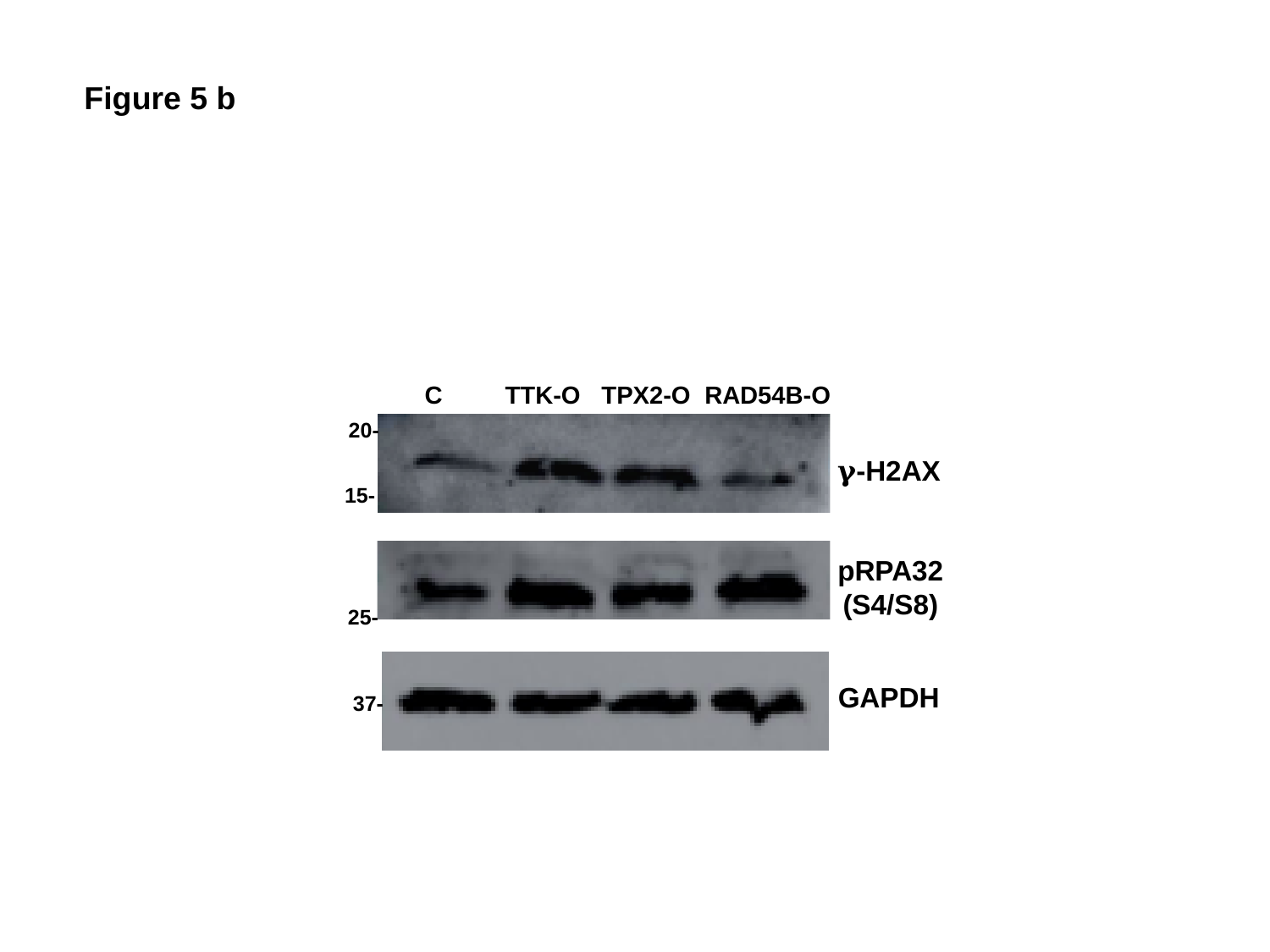

Figure 5 b
 C TTK-O TPX2-O RAD54B-O
20-
 15-
𝛄-H2AX
pRPA32 (S4/S8)
 25-
GAPDH
37-

## Slide 4
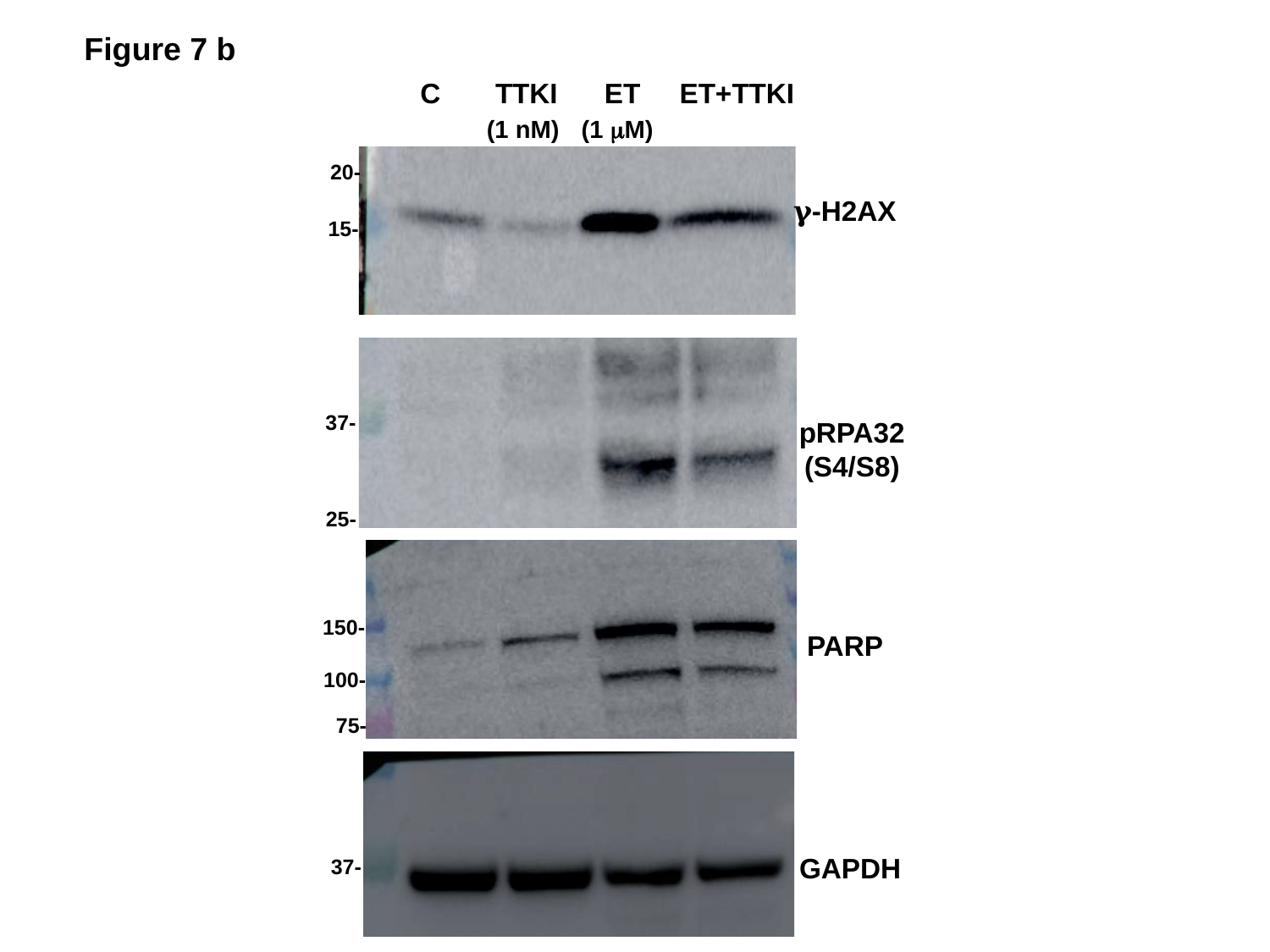

Figure 7 b
 C TTKI ET ET+TTKI
(1 nM)
(1 μM)
20-
 15-
𝛄-H2AX
37-
 25-
pRPA32 (S4/S8)
150-
 75-
PARP
100-
GAPDH
37-

## Slide 5
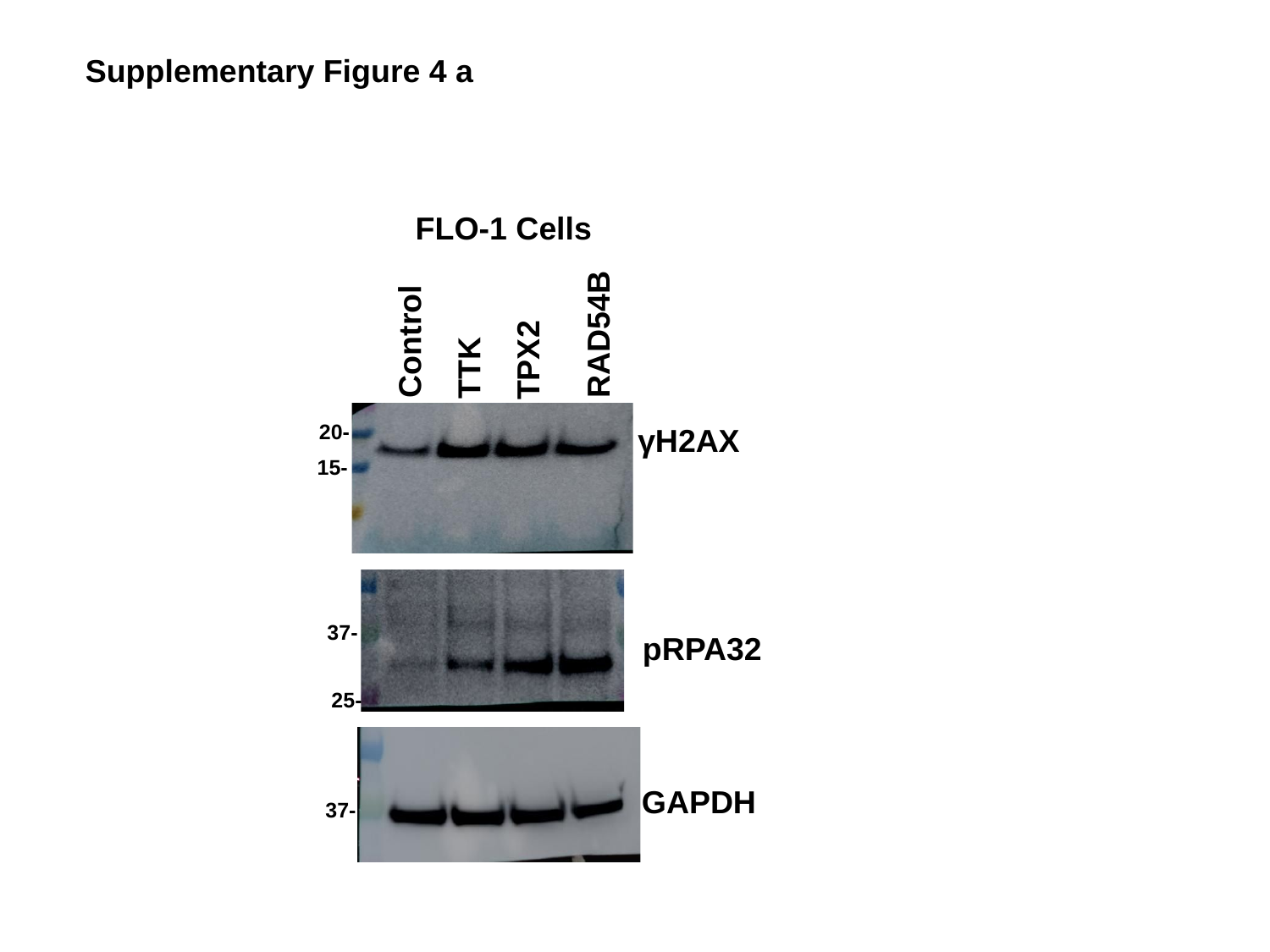

Supplementary Figure 4 a
FLO-1 Cells
RAD54B
Control
TTK
TPX2
20-
 15-
γH2AX
37-
 25-
pRPA32
GAPDH
37-
