# Supplementary material for: Integrated genomics and comprehensive validation reveal drivers of genomic evolution in esophageal adenocarcinoma
Source: Commun Biol. 2021 May 24;4:617. doi: 10.1038/s42003-021-02125-x (PMC8144613; doi:10.1038/s42003-021-02125-x)
Supplement: Supplementary file 8 — Reporting Summary [file 42003_2021_2125_MOESM8_ESM.pdf]

nature research

Corresponding author(s): MASOOD A. SHAMMAS
Last updated by author(s): 04 10 2021

Reporting Summary

Nature Research wishes to improve the reproducibility of the work that we publish. This form provides structure for consistency and transparency in reporting. For further information on Nature Research policies, see our Editorial Policies and the Editorial Policy Checklist.

Statistics

For all statistical analyses, confirm that the following items are present in the figure legend, table legend, main text, or Methods section.

n/a	Confirmed	
☐	☒	The exact sample size (*n*) for each experimental group/condition, given as a discrete number and unit of measurement
☐	☒	A statement on whether measurements were taken from distinct samples or whether the same sample was measured repeatedly
☐	☒	The statistical test(s) used AND whether they are one- or two-sided<br>*Only common tests should be described solely by name; describe more complex techniques in the Methods section.*
☒	☐	A description of all covariates tested
☒	☐	A description of any assumptions or corrections, such as tests of normality and adjustment for multiple comparisons
☐	☒	A full description of the statistical parameters including central tendency (e.g. means) or other basic estimates (e.g. regression coefficient) AND variation (e.g. standard deviation) or associated estimates of uncertainty (e.g. confidence intervals)
☐	☒	For null hypothesis testing, the test statistic (e.g. *F*, *t*, *r*) with confidence intervals, effect sizes, degrees of freedom and *P* value noted<br>*Give P values as exact values whenever suitable.*
☒	☐	For Bayesian analysis, information on the choice of priors and Markov chain Monte Carlo settings
☒	☐	For hierarchical and complex designs, identification of the appropriate level for tests and full reporting of outcomes
☐	☒	Estimates of effect sizes (e.g. Cohen's *d*, Pearson's *r*), indicating how they were calculated

Our web collection on statistics for biologists contains articles on many of the points above.

Software and code

Policy information about availability of computer code

Data collection	Genomics data has been collected from TCGA Firehose, GEO and MMRF research portal
Data analysis	R was used for general statistical analysis. Figures are generated via R core funtions, ggplot and Circos.

For manuscripts utilizing custom algorithms or software that are central to the research but not yet described in published literature, software must be made available to editors and reviewers. We strongly encourage code deposition in a community repository (e.g. GitHub). See the Nature Research guidelines for submitting code & software for further information.

Data

Policy information about availability of data

All manuscripts must include a data availability statement. This statement should provide the following information, where applicable:
- Accession codes, unique identifiers, or web links for publicly available datasets
- A list of figures that have associated raw data
- A description of any restrictions on data availability

Data availability:
1. Whole genome sequencing (WGS) data: These data have been deposited on NCBI Submission Portal. The BioProject Accession # is: PRJNA706422. The website link is:
https://dataview.ncbi.nlm.nih.gov/object/PRJNA706422?reviewer=o492if4i4odluv4ofm5p5ur8qo

2. Single nucleotide polymorphism (SNP) data: Deposited at publicly available website Harvard Dataverse.
doi = {10.7910/DVN/STUEW0}; url = {https://doi.org/10.7910/DVN/STUEW0}.

3. Supplementary documents provided with the manuscript include:
• Supplementary Material (Supplementary Figures, Table and References).
• Supplementary Data 1 (Excel file of source data underlying the graphs and charts presented in the main figures).
• Supplementary Data 2 (Full blot/gel images).
• Supplementary Data 3 (Clinical information related to esophageal adenocarcinoma (ESCA) dataset GSE19417).
• Supplementary Data 4 (Clinical information related to esophageal adenocarcinoma (ESCA) TCGA dataset).

Field-specific reporting

Please select the one below that is the best fit for your research. If you are not sure, read the appropriate sections before making your selection.

☒ Life sciences ☐ Behavioural & social sciences ☐ Ecological, evolutionary & environmental sciences

For a reference copy of the document with all sections, see nature.com/documents/nr-reporting-summary-flat.pdf

Life sciences study design

All studies must disclose on these points even when the disclosure is negative.

Sample size	For biological tests, triplicates were used to be able to measure the variance. For integrated genomics, 11 normal and 88 esophageal adenocarcinoma patient samples in The Cancer Genome Atlas were used.
Data exclusions	No data exclusion has been done.
Replication	Triplicates were used to reproduce the experiments and measure the variance.
Randomization	N/A
Blinding	N/A

Reporting for specific materials, systems and methods

We require information from authors about some types of materials, experimental systems and methods used in many studies. Here, indicate whether each material, system or method listed is relevant to your study. If you are not sure if a list item applies to your research, read the appropriate section before selecting a response.

Materials & experimental systems

n/a	Involved in the study	
☐	☒	Antibodies
☐	☒	Eukaryotic cell lines
☒	☐	Palaeontology and archaeology
☐	☒	Animals and other organisms
☒	☐	Human research participants
☐	☒	Clinical data
☒	☐	Dual use research of concern

Methods

n/a	Involved in the study	
☒	☐	ChIP-seq
☐	☒	Flow cytometry
☒	☐	MRI-based neuroimaging

Antibodies

Antibodies used	RPA2 [p Ser4, p Ser8] Antibody: Novus biologicals (NBP1-23017),<br>Phospho-Histone H2A.X (Ser139) (20E3) Cell signaling technologies (9718)<br>GAPDH (14C10) HRP Conjugate Cell signaling technologies (3683)
Validation	All the antibodies used were suggested by the manufacturers product description to react specifically with the target protein

Eukaryotic cell lines

Policy information about cell lines

Cell line source(s)	FLO-1, OE19 and OE33 were from Sigma Aldrich Corporation.<br>HEsEpiC cells were purchased from ScienCell Research Laboratories
Authentication	Cell lines were freshly purchased from Sigma and evaluated for mycoplasma.

Mycoplasma contamination	All cell lines were tested and found negative for mycoplasma.
Commonly misidentified lines (See ICLAC register)	N/A

Animals and other organisms

Policy information about studies involving animals; ARRIVE guidelines recommended for reporting animal research

Laboratory animals	CB17/Icr-Prkdcscid/IcrIcoCrl (Fox Chase SCID Mice), Female, 6 week old.
Wild animals	N/A
Field-collected samples	N/A
Ethics oversight	The protocol used for the study was approved by the Dana-Farber Cancer Institute Animal Care and Use Committee.

Note that full information on the approval of the study protocol must also be provided in the manuscript.

Clinical data

Policy information about clinical studies

All manuscripts should comply with the ICMJEguidelines for publication of clinical research and a completedCONSORT checklist must be included with all submissions.

Clinical trial registration	N/A
Study protocol	N/A
Data collection	Clinical data for genomics datasets were downloaded from Firehose or GEO.
Outcomes	Overall survival was used as an end point for the genomic datasets clinical associations.

Flow Cytometry

Plots

Confirm that:

- [x] The axis labels state the marker and fluorochrome used (e.g. CD4-FITC).
- [x] The axis scales are clearly visible. Include numbers along axes only for bottom left plot of group (a 'group' is an analysis of identical markers).
- [x] All plots are contour plots with outliers or pseudocolor plots.
- [x] A numerical value for number of cells or percentage (with statistics) is provided.

Methodology

Sample preparation	Samples for micronucleus assay were processed according to the kit protocol (MicroFlow In Vitro kit, Litron laboratories)
Instrument	BD LSR Fortessa
Software	BD FACSDiva
Cell population abundance	N/A
Gating strategy	Used the template provided by the micronucleus kit (MicroFlow In Vitro kit, Litron laboratories)

- [ ] Tick this box to confirm that a figure exemplifying the gating strategy is provided in the Supplementary Information.
